# Nomogram for Preoperative Estimation of Orbit Invasion Risk in Periocular Squamous Cell Carcinoma

**DOI:** 10.3389/fonc.2020.00564

**Published:** 2020-04-30

**Authors:** Minyue Xie, Jie Yu, Lunhao Li, Renbing Jia, Xin Song, Yefei Wang, Xianqun Fan

**Affiliations:** ^1^Department of Ophthalmology, Ninth People's Hospital, Shanghai JiaoTong University School of Medicine, Shanghai, China; ^2^Shanghai Key Laboratory of Orbital Diseases and Ocular Oncology, Shanghai, China

**Keywords:** squamous cell carcinoma, orbital invasion, nomogram, site, medial canthus

## Abstract

**Importance:** Orbital invasion occurs in some periocular squamous cell carcinoma (SCC), compromising surgical outcomes, and prognoses of patients. To date, however, there are no validation studies on the clinical features related to orbital invasion in patients with periocular SCC.

**Objective:** To explore clinical features that may be associated with orbital invasion and build a model for predicting the risk of orbital invasion.

**Design, Setting, and Participants:** In this retrospective mono-center case-control study, 90 patients with periocular SCC were treated at the Ninth People's Hospital Shanghai Jiao Tong University School of Medicine from January 2005 to August 2019. “Case” is defined as a SCC patient with orbit invasion prior to operation. “Exposure” is defined as the different sites of lesion.

**Main Outcomes and Measures:** Clinical features, including “time to relapse after surgery,” were collected. Multivariate logistic regression analysis was applied to identify the independent risk clinical features associated with orbital invasion, which was then incorporated into a nomogram.

**Results:** Of the 90 patients included in this study, 33 patients (36.7%) had orbital invasion. 14 of the 33 orbit-invasive patients had local recurrence, while 11 of 57 orbit non-invasive patients had local recurrence, suggesting that orbital invasion is a risk factor for local recurrence. The multivariate binary logistic regression indicated that the lesions at the medial canthus [odds ratio (OR), 5.024, 95% CI, 1.409–17.912, *P* = 0.013], the age at diagnosis (10-years intervals; OR, 0.590, 95% CI, 0.412–0.844, *P* = 0.004), and bleeding in the lesion (OR, 3.480, 95% CI, 1.254–9.660, *P* = 0.017) were three preoperative clinical features significantly associated with orbital invasion.

**Conclusion:** For periocular SCC, lesions at the medial canthus, the younger age of the patients at diagnosis, and bleeding in the lesion were the three main clinical features associated with orbital invasion. The risk score model for orbital invasion can act as a supportive tool for optimized clinical evaluation and treatment decisions.

## Introduction

SCC is the second most common periocular lethal tumor, usually affecting the eyelids, eyebrows, and canthus. It accounts for 5%−10% of all eyelid malignancies (1–6). It ranks after basal cell carcinoma (BCC) in periocular tumors but is more aggressive than BCC (2). SCC is commonly the result of prolonged ultraviolet radiation exposure and sometimes progresses from actinic keratoses (7). Therefore, SCC is more common in people with light skin, and incidence increases with age and geographic distance from the equator. Such tumors usually present as a scaly hard lump or a flat sore on an old scar. When SCC occurs in periocular skin, it may spread into the orbit or metastasize to the regional lymph nodes, leading to significant mortality if not treated in time (1, 8). Although the mortality of periocular SCC is low overall, extensive orbit invasive SCC may lead to the patient having to undergo orbital exenteration, which greatly affect the patient's vision and quality of life (9). Moreover, orbit invasion means a higher T stage, which has been shown to be significantly associated with local recurrence in a previous study (10). In addition, the presence of orbit invasion is a histopathologic feature that suggests aggressive behavior of the SCC and usually shows a worse prognosis after operation. Currently, the diagnosis of orbit invasion is determined upon image examination of patients with significant orbital invasion, and for patients with insignificant orbital invasion, postoperative histologic examination is needed. Accurate estimation for the presence of orbit invasion before surgery can help surgeons to choose appropriate surgical approach. Also, high-risk patients need more radical management and closer follow-ups. In this context, we aimed to explore clinical characteristics related to orbital invasion. However, there are no studies on the clinical characteristics related to orbital invasion to date. Thus, it is necessary to explore the clinical features associated with orbit invasion and pay close attention to high-risk patients.

Previous studies in Caucasian populations have shown that, in order of frequency, SCC occurs primarily on the lower eyelid followed by the medial canthus, upper eyelid, the lateral canthus, and the brow (11, 12). However, the frequency of SCC lesion sites in Asian populations has not been demonstrated. It is also not known whether the lesion site in periocular SCC is associated with the risk of orbit invasion. The identification of correlations between orbital invasion and clinical features, such as lesion site, will have significance in guiding whether further imaging examinations should be performed and the choice of surgical methods.

It is necessary to develop a prediction model based on preoperative clinical data incorporating factors related to orbit invasion. Among all available models, nomograms can provide evidence-based and personalized risk estimates facilitating management-related decisions and prognosis assessment. Thus, based on this model, our intent was to investigate the clinical features potentially associated with orbit invasion in patients with periocular SCC.

## Methods

### Patients

A case-control study was applied to determine the relationship of orbit invasion with clinical features. The patients involved in this study were identified through a search for the terms “SCC” and “ophthalmology department” in the pathologic database at Ninth People's Hospital Shanghai Jiao Tong University School of Medicine between January 2005 and August 2019.

The inclusion criteria included: (1) patient with periocular masses, and (2) postoperative pathological findings of squamous cell carcinoma. Ninety-five patients with periocular skin SCC were identified. Five patients with conjunctival SCC were excluded, leaving a final sample size of ninety patients. This research was approved by the Ethics Committee of the Ninth People's Hospital affiliated with Shanghai Jiao Tong University School of Medicine. We obtained informed consent from all patients to use their data for research. Patients did not receive financial compensation.

### Clinical Variables and Tumor-Related Variables

**Figure 1:**
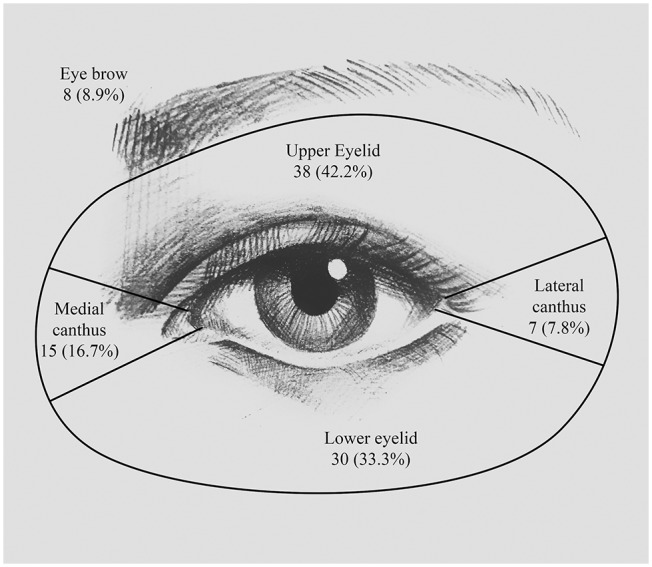
Location of periocular SCC. Numbers and percentages of patients having lesion at the upper eyelid, the lower eyelid, the medial canthus, the eyebrow, and the lateral canthus.

The clinical variables involved in this study were presented in Table 1. The data collected included age, sex, laterality, HBV infection, diabetes, blood glucose, hypertension, site of the lesion (as outlined in Figure 1), and lesion state. Other tumor-related data, such as surgical approach, perineural invasion (PNI), and pathological grade, were also reported (Table 1). All surgical specimens underwent routine histopathological examinations performed independently by two pathologists. Whether orbit invasion occurred was determined based on postoperative pathological results.

**Table 1:** The demographic and clinical features of patients with orbital invasion and non-invasion.

	**Total, *n* = 90**	**Orbit invade, *n* = 33 (36.7%)**	**Non-invasive, *n* = 57 (63.3%)**	***P*-value**
Gender	–	–	–	0.895
Male	51 (56.7%)	19 (57.6%)	32 (56.1%)	–
Female	39 (43.3%)	14 (42.4%)	25 (43.9%)	–
Age (year)	68.78 ± 14.37	62.94 ± 15.13	72.16 ± 12.87	0.003
Laterality	–	–	–	0.801
Left	53 (58.9%)	20 (60.6%)	33 (57.9%)	–
Right	37 (41.1%)	13 (39.4%)	24 (42.1%)	–
Diabetes	10 (11.1%)	3 (9.1%)	7 (12.3%)	0.643
Blood glucose	5.37 ± 1.43	5.45 ± 1.57	5.37 ± 1.43	0.580
Hypertension	18 (20.0%)	7 (21.2%)	11 (19.3%)	0.827
HBV	–	–	–	0.805
HBsAg (–)	62 (84.9%)	25 (86.2%)	37 (84.1%)	–
HBsAg (+)	11 (15.1%)	4 (13.8%)	7 (15.9%)	–
Site	–	–	–	–
Upper eyelid	38 (42.2%)	8 (24.2%)	30 (52.6%)	0.009
Lower eyelid	30 (33.3%)	13 (39.4%)	17 (29.8%)	0.353
Medial canthus	15 (16.7%)	10 (30.3%)	5 (8.8%)	0.008
Brow	8 (8.9%)	1 (3.0%)	7 (12.3%)	0.250
Lateral canthus	7 (7.8%)	2 (6.1%)	5 (8.8%)	0.712
Bleeding in the lesion	29 (32.2%)	13 (22.8%)	16 (48.5%)	0.012
Perineural invasion	13 (14.4%)	7 (21.2%)	6 (10.5%)	0.165
Surgical approach	–	–	–	<0.01
Wide local excision	21 (23.3%)	3 (9.1%)	18 (31.6%)	–
MMS	47 (52.2%)	8 (24.2%)	39 (68.4%)	–
Orbital exenteration	22 (24.4%)	22 (66.7%)	0 (0%)	–

*Continuous parameters presented as mean ± SD, categorical data as n (%)*.

### Statistical Analysis

The analyses were performed using SPSS (version 24.0, IBM, Armonk, NY) and R version 3.6.1 (The R Foundation). We reported the frequency (percentage) for categorical variables, mean (standard deviation) for normal-distributed continuous variables, and median (quartile) for variables without normal distribution. We first analyzed the clinical features of patients with and without orbit invasion by chi-square tests (categoric variables). For continuous variables, student *t*-tests were applied if variables were normally distributed while Kruskal-Willus tests were used for highly-skewed variables. In order to determine the association of recurrence with orbit invasion and other factors in patients with periocular SCC, we also calculated the hazard ratios (HRs) and 95% confidence intervals (CIs) using cox regression models. A value of *P* < 0.05 was considered significant.

To explore the independent impact of the clinical characteristics for orbit invasion by SCC, the significance of each variable was assessed by univariate logistic regression analysis. All variables significantly associated with orbit invasion were suitable for stepwise multivariate analysis. We then analyzed lesion location, age at diagnosis (10-years intervals), and lesion bleeding as the three most likely risk factors for orbit invasion by using multivariate binary logistic regression models. We calculated the ORs and 95% CIs, and a value of *P* < 0.05 was considered significant. Based on the multivariate logistic regression analysis results, a nomogram was formulated using R's rms package version 3.0. The nomogram scaled each regression coefficient based on multivariate logistic regression to a scale of 0–100 points. The variable with the highest absolute value of the β coefficient was assigned 100 points. Independent variable points were added to get the total points and a predicted probability was converted. The prediction performance was measured by the consistency index (C index) and calibrated with 1,000 bootstrap samples to reduce the overfitting bias.

## Results

### Patients, Tumor Characteristics, and Treatment Data

Among the 90 patients enrolled in this study, 51 were male (56.7%) and 39 were female (43.3%). The average age of diagnosis was 68.78 years, whereas the median age was 69 years (range 24–100 years) (Table 1). The average age of the male patients was 66.98 (±14.45) years, while female patients had an average age of 71.13 (±14.11) years. However, this sex-related age difference was not statistically significant (*P* = 0.175). Orbit invasion occurred in 19 (57.6%) male patients and 14 (42.4%) female patients. There was no statistical relationship between gender and orbital invasion (*P* = 0.895).

In this study, SCC seemed to have a higher probability of affecting the left eyelid [53 patients (58.9%)] than the right eyelid [37 patients (41.1%)], but this difference was not statistically significant (*P* = 0.092). The locations of the lesions are detailed in Figure 1. The most common site was the upper eyelid [38 patients (42.2%)], followed by the lower eyelid [30 patients (33.3%)] and the medial canthus [15 patients (16.7%)]. The remaining few patients developed lesions at the eyebrow [8 patients (8.9%)] and the lateral canthus [7 patients (7.8%)]. We have found that out of the 38 patients who had lesions on the upper eyelid, orbit invasion occurred in only 8 patients (24.2%) (*P* = 0.009). While SCCs that occurred in the medial canthus were more prone to orbit invasion; for example, of the 15 patients (16.7%) who had lesions on the medial canthus, 10 patients (30.3%) suffered orbit invasion (*P* = 0.008).

Ten patients (11.1%) had diabetes, of which 3 patients (9.1%) had orbit invasion, while the other 7 patients (12.3%) were without orbit invasion (*P* = 0.643). Average blood glucose on the first day of admission was 5.37 (± 1.43). In patients with orbit invasion, the average blood glucose was 5.45 (± 1.57), while the average blood glucose of patients without orbit invasion was 5.37 (± 1.43). This difference was not statistically significant (*P* = 0.580). 18 patients (20.0%) had hypertension, of which 7 patients (21.2%) had orbit invasion, while the other 11 patients (19.3%) were without orbit invasion (*P* = 0.827). 11 patients (15.1%) showed hepatitis B virus (HBV) infection, based on the fact of the hepatitis B surface antigen (HbsAg) positive. Four (13.8%) of these 11 patients suffered orbit invasion (*P* = 0.805).

Thirteen patients (14.4%) who presented with severe pain had histologic PNI, of which 3 patients had lesions on the upper eyelid, one had lesions on both the upper and lower eyelids, one had a lesion on the medial canthus, and one has a lesion on the lateral canthus. Nine of these 13 patients had well differentiated SCCs, two patients had moderately differentiated, and the other two patients were poorly differentiated. Seven patients (21.2%) had orbit invasion, and six patients (10.5%) were without orbit invasion. PNI had no significant correlation with the risk of orbit invasion (*P* = 0.165).

Local treatment of SCC includes wide local excision, Mohs micrographic surgery (MMS) and orbital exenteration. 21 patients (23.3%) underwent wide local excision, of which 3 patients (9.1%) had orbit invasion and 18 patients (31.6%) were without orbit invasion. 47 patients (52.2%) underwent MMS, of whom 8 (24.2%) had exhibited orbit invasion and 39 (68.4%) were without orbit invasion. The remaining 22 patients (24.4%) underwent orbital exenteration, all of whom (66.7%) had orbit invasion. According to our observations, orbit invasion was significantly associated with the choice of surgical treatment (*P* < 0.01).

### Postoperative Prognosis and Independent Prognostic Factors

After a median follow-up of 49 months (range, 6–180 months), among the 33 patients who had orbit invasion, 19 patients remained relapse free, while 14 patients had local recurrence; this suggests that patients with orbit invasion had a higher risk of local recurrence (HR, 2.269, 95% CI, 1.029–5.001, *P* = 0.042). We then analyzed the relevance between orbit invasion and local recurrence by univariate Cox regression analysis (Supplementary Figure 1). The estimated recurrence-free survival time of patients with or without orbit invasion was 130.929 (111.486–150.372) months and 88.475 (65.240–111.674) months, respectively.

### Prediction Model for Orbit Invasion

All variables involved in this analysis were derived from preoperative data. The tumor-related clinical features, including lesion site, lesion state, and whether it is accompanied by pain were assessed before surgery.

After analyzing the clinical features by chi-square tests (categoric variables) and independent sample *t* tests (continuous variables), clinical features with a *P* < 0.2 were included as predictors in the multivariate predictive model. The final multivariable model included lesions at the medial canthus (OR, 4.947, 95% CI, 1.377–17.778, *P* = 0.014), the age at diagnosis (by 10-years interval, OR, 3.422, 95% CI, 1.286–9.103, *P* = 0.014), and bleeding in the lesion (OR, 3.005, 95% CI, 1.107–8.156, *P* = 0.031) as the three clinical features significantly associated with orbit invasion (Table 2).

**Table 2:** The multivariate logistic regression analysis of orbital invasion.

	**Odds ratio (95%CI)**	***P*-value**
Age/10	0.590 (0.412–0.844)	0.004
Bleeding in the lesion	3.480 (1.254–9.660)	0.017
Medial canthus lesion	5.024 (1.409–17.912)	0.013

**Figure 2:**
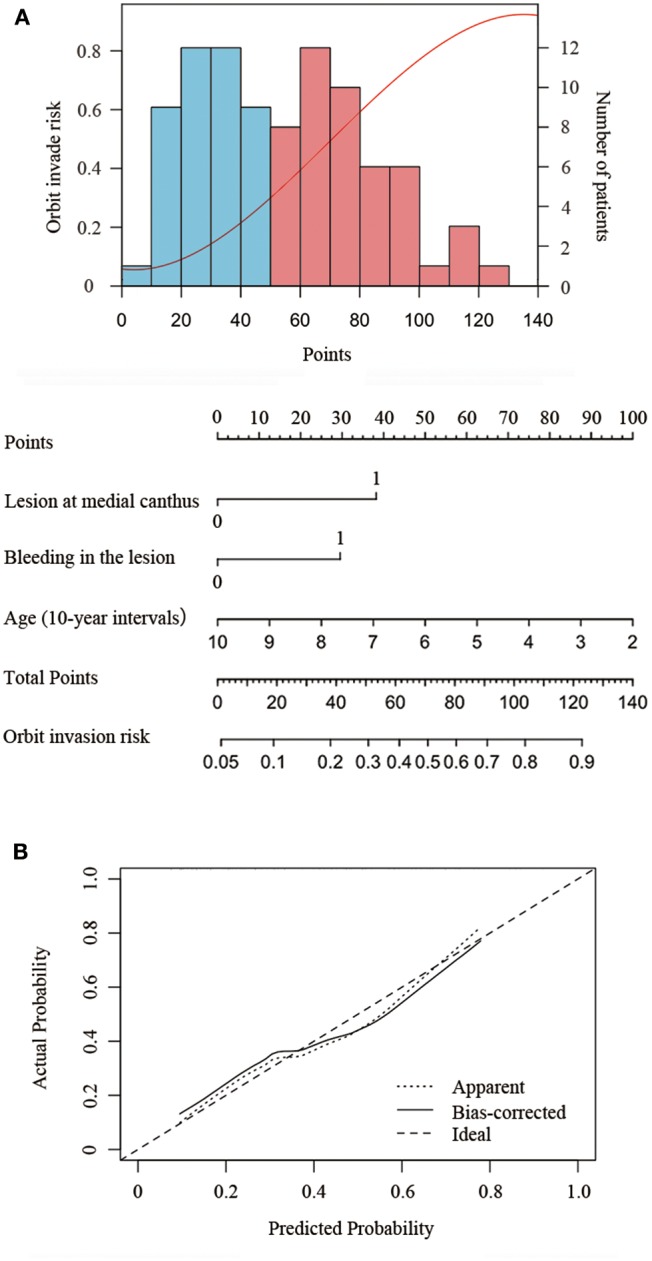
Nomogram to predict the risk of orbit invasion in patients of periocular SCC. **(A)** Risk curve refers to the orbit invasion possibility based on the risk scores of different risk factors. Histogram shows the distribution of risk factors scores in this cohort. The blue bar refers to low risk and the red bars refers to high risk. Instruction of the nomogram: Draw a vertical line from the end of the axis of each risk factor to the top line labeled “Points.” Sum up the points of the three risk factors as total points. Then draw a vertical line down from the axis labeled “Total Points” to the bottom line labeled “Orbit invasive risk” to get the predicted possibility of orbit invasion. **(B)** The calibration plot showed the actual probability of orbit invasion over the predicted risk probability.

The nomogram developed from the final multivariable model for predicting SCC orbit invasion is shown in Figure 2A. This three-item orbit-invasive risk score (age, lesion bleeding, and lesion site) assigned each factor a corresponding point value based on the correlation of each predictor with orbit invasion. The total risk points could be calculated by adding up the points of each risk factor, which could be obtained by drawing a vertical line to the “Points” axis. Then the predicted possibility of orbit invasion could be obtained by drawing a vertical line down from the “Total Points” axis to the “Orbit invasive risk” axis. The bootstrap validation method was used to validate the resulting model internally. The nomogram demonstrated relatively good performance in estimating the orbit invasion risk, with a C index of 0.77. In addition, the calibration plot graphically showed the consistency of orbit invasion between the risk predicted by the nomogram and actual risk confirmed by histopathologic examination (Figure 2B).

Patients with fewer than 30 points were considered to have low risk of orbit invasion. Patients with points between 30 and 75 points were medium-risk patients and were recommended to take orbit CT and MRI to exclude the possibility of orbital invasion. If orbital invasion is confirmed, ultrasonography of the cervical lymph nodes and parotid gland as well as chest CT and abdominal ultrasonography were recommended to exclude the possibility of parotid or lymph node metastases and distant metastases. Patients scoring higher than 75 points were at high risk. Thus, orbital CT and MRI, ultrasonography of cervical lymph nodes and parotid gland, as and a chest CT and abdominal ultrasonography were recommended to guide the choice of surgical approach (Supplementary Figure 2).

## Discussion

This report constitutes the largest retrospective series of Chinese patients with periocular SCC. We found an orbit invasion rate of 36.7%, which was not very low in light of the low risk of SCC progressing to invasive status. Since a considerable number of patients were from rural areas, the economic level was low, there was a lack of health awareness, and delays in treatment were very common; patients often ignored the eyelid lesions until they developed severe symptoms, such as bleeding or pain, or eyelid function was affected. As of this writing, SCC is the most common secondary epithelial tumor in the orbit (13). SCC can be cured by surgical resection if it is diagnosed early. However, to cure orbit-invading SCC, orbital exenteration is often required.

### Clinical Factors

In this study, we found the top three affected sites of SCC to be the upper eyelid (42.2%), the lower eyelid (33.3%), and the medial canthus (16.7%). The periocular distribution of SCC has always been a controversial topic. Previous reports have found that the site with the highest incidence of SCC was the lower eyelid, followed by the upper eyelid or the medial canthus (2, 3, 11). The difference in findings between our report and previously published studies may be related to the difference in the orbit structure between Asians and Caucasians. Caucasians tend to have deeper and wider orbits than Asians, and UV exposure has been proven to be a risk factor for SCC. The superior orbital rim projects more anteriorly in Caucasians. Thus, the upper eyelids of Asians tend to be more prominent, leading to greater direct solar exposure. This is also consistent with higher incidence, although not statistically significant, of periocular SCC on the left side in the Chinese population in the present study. In China, vehicles drive on the right side of road, which causes the left side of drivers to be more exposed to UV rays. However, single-center-based statistics for epidemiological studies may not be fully reliable due to the low incidence of SCC.

From our observation, the mean age of diagnosis was 68.78 years, which was slightly older than the average age of 66 years found in Australia and America (3, 12). This may be related to the fact that few Chinese people have tanning habits, which cause additional UV exposure.

### Orbit Invasion

SCC is usually a non-lethal cancer, although when left untreated SCC could be aggressive and spread to nearby healthy tissue, causing serious complications (14). When the cancer is particularly large and deep or when it involves the mucous membranes, the risk of aggressive SCC is increased (15). As SCC progresses, it can grow continuously and can be accompanied by crusting or bleeding. We found that patients with bleeding in the lesion, which quite likely suggested a more aggressive SCC, had a greater risk of orbit invasion. We also found that the lesion site was related to the subclinical tumor extension of periocular SCC. Tumors that occurred at the medial canthus, which implied a greater possibility of involving the mucous membranes, were more likely to invade the orbit. The greatest risk factor for SCC was the total amount of exposure to UV radiation (16), so it is not difficult to understand that SCC mostly occurs in populations over 60 years old (17). From our observation, although patients under 63 years of age are less likely affected by SCC, they tend to have more aggressive SCC when affected, which may be the reason that individuals of younger age were more prone to orbital invasion. However, few years of age difference is clinically less meaningful, therefore, we chose to evaluate age at diagnosis in 10-years intervals to obtain a more clinically meaningful factor.

Previous research has suggested that human papillomavirus (HPV) infection is associated with cutaneous SCC (18, 19). Patients with the presence of PNI, with chronic immunosuppression and poor differentiation, proved to have high-risk lesions (20–23). Unfortunately, due to the insufficient sample size in our study, we failed to observe the relationship between viral infection, PNI, and immunosuppressive status and orbital invasion.

In the present study, we have not observed the correlation between diabetes and orbit invasion in periocular SCC due to the sample size limitations. But a recent study on cutaneous SCC has suggested that diabetes was a risk factor for recurrence in the Asian population (24). As diabetes is an immunocompromised state caused by hyperglycemia (25, 26), it is therefore associated with an increased risk of various malignancies. Thus, we hypothesize that there may be a potential association between diabetes and periocular SCC; we hope for this thesis to be confirmed in future studies of larger sample size.

Since only an estimated 3%−8% of untreated SCC *in situ* progresses to invasive SCC and most SCC lesions are limited to the eyelids (27), imaging examination of the orbit is not a routine examination item for patients with suspected SCC. Thus, the diagnosis of orbit-invading SCC mostly depends on the doctor's experience. To more reliably predict the possibility of orbit invasion, we developed and validated a new predictive tool using three easily accessible variables. This risk score can help doctors assess the risk of orbit invasion using clinical features. For patients deemed to be at high risk of orbit invasion, choosing to perform an imaging examination of the orbit might help to identity early orbit invasion and guide the choice of surgical approach and follow-up treatment. Furthermore, this risk score can also help doctors focus on prompting patients with the clinical features of risk factors when conducting health education.

Using the nomogram to estimate the risk of patients harboring orbit invasion and to guide clinical treatment is a new concept. Because orbit invasive status is not the only factor determining the therapeutic procedures of periocular SCC, other factors that are not included in this model, such as the overall performance of patients, whether the patient is immunosuppressed, and tumor size, should also be considered.

### Study Limitations

The current research still has a few limitations. SCC arising in scars and in chronically immunosuppressed patients might indicate a risk of orbit invasion, but due to the small number of such cases, the risk score could not be accurately calculated. In addition, the viral infection and underlying diseases, including diabetes and hypertension, were also not statistically significant due to the small sample size and lack of data. Furthermore, this study only included the patients from the Chinese population; thus, the generalizability of our results to other populations outside China is uncertain. Evaluation in a wider SCC population is still necessary. Finally, all data used in this analysis was from a single institution; it is necessary to verify for similar results from other centers.

## Conclusion

In summary, orbit invasion in periocular SCC usually suggests a more aggressive lesion and a worse prognosis. Therefore, we suggest a routine calculation of orbit invasion risk score when evaluating patients with suspected SCC. For high-risk patients, imaging examination of the orbit is recommended, and appropriate treatment as well as closer follow-up should be performed. The risk score model for orbital invasion can act as a supportive tool for optimized clinical evaluation and treatment decisions.

## Data Availability Statement

All datasets generated for this study are included in the article/Supplementary Material.

## Ethics Statement

The studies involving human participants were reviewed and approved by Ethics Committee of the Ninth People's Hospital affiliated with Shanghai Jiao Tong University School of Medicine. Written informed consent for participation was not required for this study in accordance with the national legislation and the institutional requirements.

## Author Contributions

XF, YW, and XS supervised the project and provided direction and guidance throughout the preparation of this manuscript. MX, JY, and LL extracted all data and performed the analyses. MX drafted the paper. All authors contributed to and revised the final manuscript.

## Conflict of Interest

The authors declare that the research was conducted in the absence of any commercial or financial relationships that could be construed as a potential conflict of interest.

## Supplementary Material

Supplementary Figure 1 Recurrence-free survival in patients with or without orbit invasion using Cox regression univariate analysis (*P* = 0.042).

Supplementary Figure 2 The decision tree visualized the different recommendation for further examination and treatment based on the risk score.

## Abbreviations

BCC: basal cell carcinoma
C index: consistency index
CI: confidence intervals
HPV: human papillomavirus
HR: hazard ratio
OR: odds ratio
PNI: perineural invasion
SCC: squamous cell carcinoma.

## References

[1] Cook BE Bartley GB. Treatment options and future prospects for the management of eyelid malignancies: an evidence-based update. Ophthalmology. (2001) 108:2088-98; quiz 2099-2100, 2121. 10.1016/S0161-6420(01)00796-5 11713084

[2] Faustina M Diba R Ahmadi MA Esmaeli B. Patterns of regional and distant metastasis in patients with eyelid and periocular squamous cell carcinoma. Ophthalmology. (2004) 111:1930–2. 10.1016/j.ophtha.2004.02.009 15465559

[3] Malhotra R Huilgol SC Huynh NT Selva D. The Australian Mohs database: periocular squamous cell carcinoma. Ophthalmology. (2004) 111:617–23. 10.1016/j.ophtha.2003.07.020 15051191

[4] Limawararut V Leibovitch I Sullivan T Selva D. Periocular squamous cell carcinoma. Clin Experiment Ophthalmol. (2007) 35:174–85. 10.1111/j.1442-9071.2006.01411.x 17362462

[5] Nasser QJ Roth KG Warneke CL Yin VT El Sawy T Esmaeli B. Impact of AJCC 'T' designation on risk of regional lymph node metastasis in patients with squamous carcinoma of the eyelid. Br J Ophthalmol. (2014) 98:498–501. 10.1136/bjophthalmol-2013-304434 24429279

[6] Fu T Aasi SZ Hollmig ST. Management of high-risk squamous cell carcinoma of the skin. Curr Treat Options Oncol. (2016) 17:34. 10.1007/s11864-016-0408-2 27262708

[7] Borden ES Kang P Natri HM Phung TN Wilson MA Buetow KH. Neoantigen fitness model predicts lower immune recognition of cutaneous squamous cell carcinomas than actinic keratoses. Front Immunol. (2019) 10:2799. 10.3389/fimmu.2019.02799 31849976 PMC6896054

[8] Yanagi T Kitamura S Hata H. Novel therapeutic targets in cutaneous squamous cell carcinoma. Front Oncol. (2018) 8:79. 10.3389/fonc.2018.00079 29629337 PMC5876309

[9] Zhang Z Ho S Yin V Varas G Rajak S Dolman PJ. Multicentred international review of orbital exenteration and reconstruction in oculoplastic and orbit practice. Br J Ophthalmol. (2018) 102:654–8. 10.1136/bjophthalmol-2017-310681 28844052

[10] Sun MT Andrew NH O'Donnell B McNab A Huilgol SC Selva D. Periocular squamous cell carcinoma: TNM staging and recurrence. Ophthalmology. (2015) 122:1512–6. 10.1016/j.ophtha.2015.04.002 25972255

[11] Cook BE Bartley GB. Epidemiologic characteristics and clinical course of patients with malignant eyelid tumors in an incidence cohort in Olmsted County, Minnesota. Ophthalmology. (1999) 106:746–50. 10.1016/S0161-6420(99)90161-6 10201597

[12] Xu S Sagiv O Rubin ML Sa HS Tetzlaff MT Nagarajan P. Validation study of the AJCC cancer staging manual, eighth edition, staging system for eyelid and periocular squamous cell carcinoma. JAMA Ophthalmol. (2019) 137:537–42. 10.1001/jamaophthalmol.2019.0238 30869769 PMC6512305

[13] Soysal HG Markoç F. Invasive squamous cell carcinoma of the eyelids and periorbital region. Br J Ophthalmol. (2007) 91:325–9. 10.1136/bjo.2006.102673 17020898 PMC1857650

[14] Brantsch KD Meisner C Schönfisch B Trilling B Wehner-Caroli J Röcken M. Analysis of risk factors determining prognosis of cutaneous squamous-cell carcinoma: a prospective study. Lancet Oncol. (2008) 9:713–20. 10.1016/S1470-2045(08)70178-5 18617440

[15] Stratigos A Garbe C Lebbe C Malvehy J del Marmol V Pehamberger H. Diagnosis and treatment of invasive squamous cell carcinoma of the skin: european consensus-based interdisciplinary guideline. Eur J Cancer. (2015) 51:1989–2007. 10.1016/j.ejca.2015.06.110 26219687

[16] Gandhi SA Kampp J. Skin cancer epidemiology, detection, and management. Med Clin North Am. (2015) 99:1323–35. 10.1016/j.mcna.2015.06.002 26476255

[17] Korhonen N Ylitalo L Luukkaala T Itkonen J Häihälä H Jernman J. Characteristics and trends of cutaneous squamous cell carcinoma in a patient cohort in finland 2006-2015. Acta Derm Venereol. (2019) 99:412–6. 10.2340/00015555-3110 30628632

[18] Pfister HJ Gariglio M Smola S. Editorial: human papillomaviruses and polyomaviruses in skin cancer. Front Microbiol. (2018) 9:2778. 10.3389/fmicb.2018.02778 30498488 PMC6250093

[19] Purdie KJ Proby CM Rizvi H Griffin H Doorbar J Sommerlad M. The role of human papillomaviruses and polyomaviruses in BRAF-inhibitor induced cutaneous squamous cell carcinoma and benign squamoproliferative lesions. Front Microbiol. (2018) 9:1806. 10.3389/fmicb.2018.01806 30154763 PMC6102365

[20] Burton KA Ashack KA Khachemoune A. Cutaneous squamous cell carcinoma: a review of high-risk and metastatic disease. Am J Clin Dermatol. (2016) 17:491–508. 10.1007/s40257-016-0207-3 27358187

[21] Thompson AK Kelley BF Prokop LJ Murad MH Baum CL. Risk factors for cutaneous squamous cell carcinoma recurrence, metastasis, and disease-specific death: a systematic review and meta-analysis. JAMA Dermatol. (2016) 152:419–28. 10.1001/jamadermatol.2015.4994 26762219 PMC4833641

[22] Karia PS Morgan FC Ruiz ES Schmults CD. Clinical and incidental perineural invasion of cutaneous squamous cell carcinoma: a systematic review and pooled analysis of outcomes data. JAMA Dermatol. (2017) 153:781–8. 10.1001/jamadermatol.2017.1680 28678985 PMC5657475

[23] Que SKT Zwald FO Schmults CD. Cutaneous squamous cell carcinoma: Incidence, risk factors, diagnosis, and staging. J Am Acad Dermatol. (2018) 78:237–47. 10.1016/j.jaad.2017.08.059 29332704

[24] Oh Y Kim J Zheng Z Kim SK Chung KY Roh MR. Risk factors for recurrence in cutaneous squamous cell carcinoma after Mohs micrographic surgery: a retrospective review of 237 Asian patients. J Dermatol. (2020) 47:72–7. 10.1111/1346-8138.15129 31674043

[25] Delamaire M Maugendre D Moreno M Le Goff MC Allannic H Genetet B. Impaired leucocyte functions in diabetic patients. Diab Med. (1997) 14:29–34 10.1002/(SICI)1096-9136(199701)14:1<29::AID-DIA300>3.0.CO;2-V 9017350

[26] Boyanova L Mitov I. Antibiotic resistance rates in causative agents of infections in diabetic patients: rising concerns. Expert Rev Anti Infect Ther. (2013) 11:411–20. 10.1586/eri.13.19 23566150

[27] Eimpunth S Goldenberg A Hamman MS Oganesyan G Lee RA Hunnangkul S. Squamous cell carcinoma in situ upstaged to invasive squamous cell carcinoma: a 5-year, single institution retrospective review. Dermatol Surg. (2017) 43:698–703. 10.1097/DSS.0000000000001028 28060173

