# Supplementary figures and images for: Nomogram for Preoperative Estimation of Orbit Invasion Risk in Periocular Squamous Cell Carcinoma
Source: Front Oncol. 2020 Apr 30;10:564. doi: 10.3389/fonc.2020.00564 (PMC7203342; doi:10.3389/fonc.2020.00564)

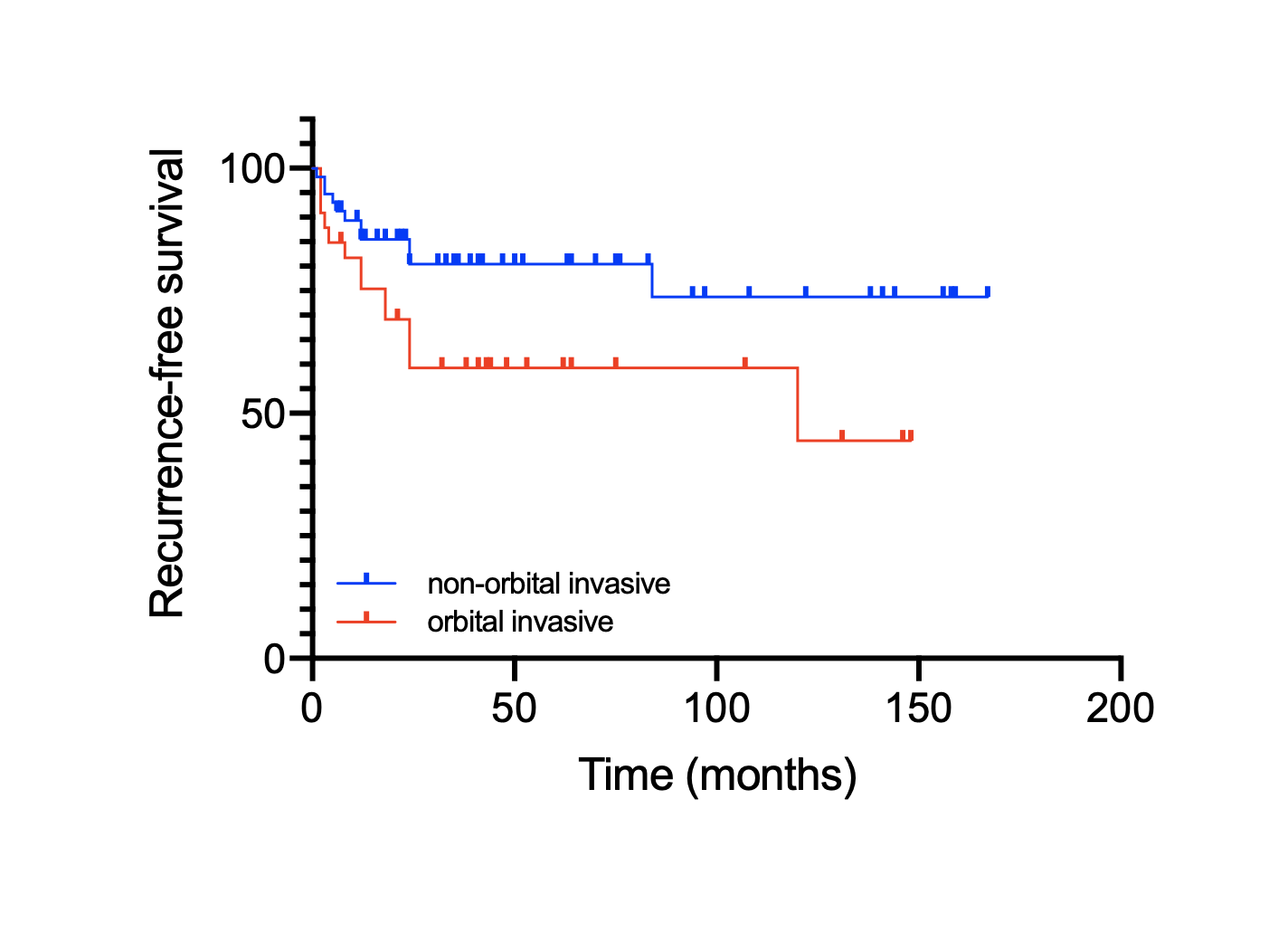

Supplement: Supplementary Figure 1 — Recurrence-free survival in patients with or without orbit invasion using Cox regression univariate analysis (P = 0.042). [file Image_1.TIF]

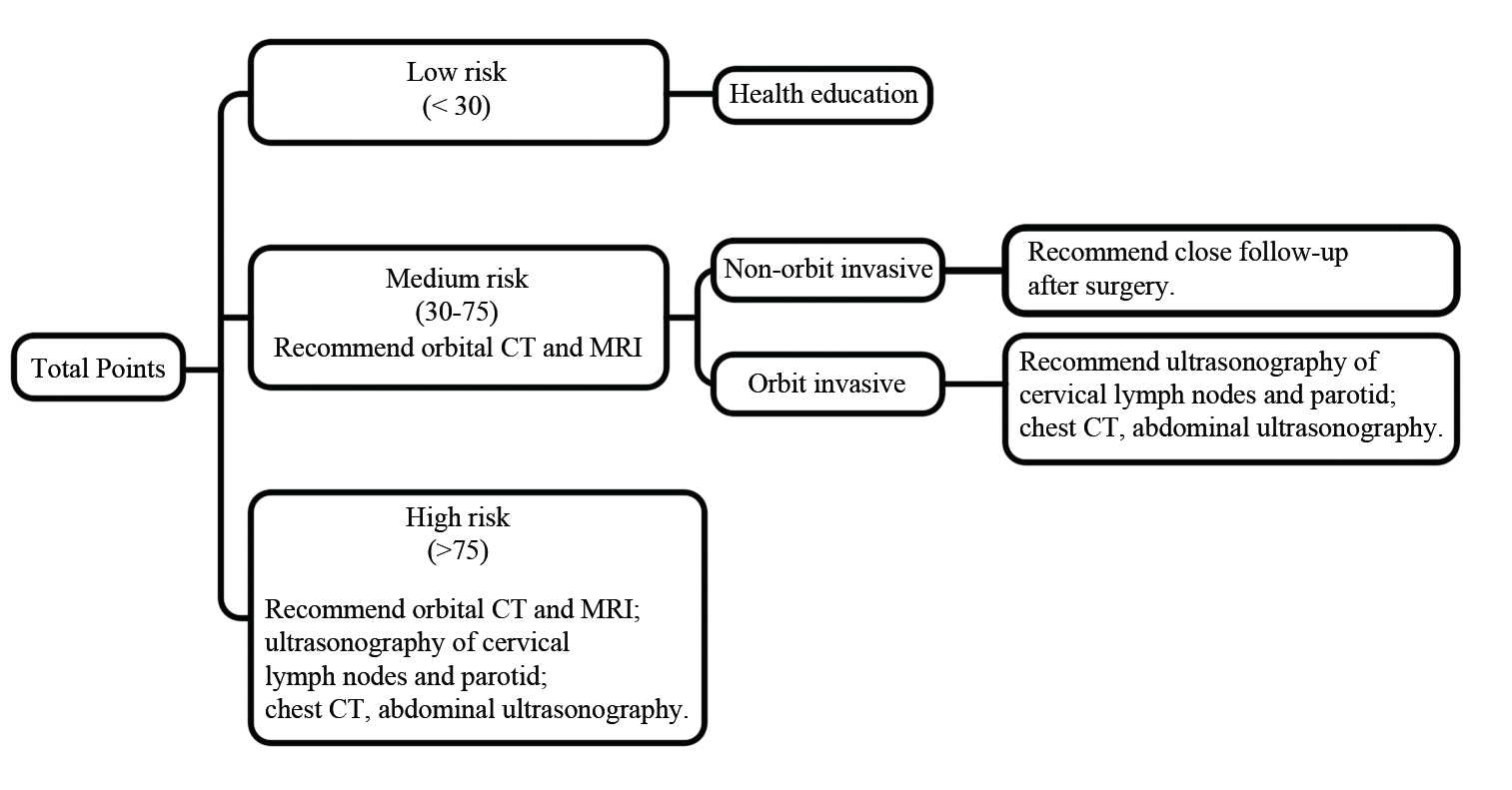

Supplement: Supplementary Figure 2 — The decision tree visualized the different recommendation for further examination and treatment based on the risk score. [file Image_2.TIF]
